# Computed tomography versus aortography for transcatheter patent ductus arteriosus closure in adults

**DOI:** 10.1007/s10554-025-03515-6

**Published:** 2025-09-29

**Authors:** Takashi Miki, Toru Miyoshi, Teiji Akagi, Mitsutaka Nakashima, Rie Nakayama, Yoichi Takaya, Koji Nakagawa, Norihisa Toh, Shinsuke Yuasa

**Affiliations:** Department of Cardiovascular Medicine, Dentistry and Pharmaceutical Sciences, Okayama University Graduate School of Medicine, 2-5-1 Kitaku Shikata-cho, Okayama, 700-8558 Japan

**Keywords:** Patent ductus arteriosus, Computed tomography, Aortography, Transcatheter closure

## Abstract

Accurate sizing of the patent ductus arteriosus (PDA) is essential for successful transcatheter closure. While aortography is the standard imaging modality, computed tomography (CT) may offer superior anatomical visualization. This study aimed to compare the accuracy and procedural outcomes of preprocedural CT versus aortography alone in adult patients undergoing transcatheter PDA closure. We retrospectively analyzed 54 adult patients who underwent PDA closure using the Amplatzer™ Duct Occluder between 2009 and 2024. Nineteen patients were treated based on aortography alone and 35 based on preprocedural CT. We compared procedural characteristics and outcomes, including device size exchange and procedure time. A simulation study was also conducted in which two blinded implanters independently predicted occluder size based on CT and aortography, with actual implanted device size used as the reference. The CT group had significantly larger PDA sizes and implanted device sizes. Device replacement was required in three patients in the aortography group but none in the CT group. Procedure time was shorter in the CT group (60 ± 9 vs. 70 ± 14 min, *p* = 0.003). Simulation results showed that CT more accurately predicted the actual implanted device size (85% vs. 63%, *p* = 0.008). PDA size at the pulmonary artery end was significantly underestimated by aortography. Preprocedural CT improved procedural efficiency and device selection accuracy in adult PDA closure. These findings suggest that CT imaging may enhance planning and safety in transcatheter PDA interventions.

## Introduction

Patent ductus arteriosus (PDA) is a congenital cardiovascular anomaly characterized by persistent patency of the fetal ductus arteriosus after birth [1]. Although it is commonly diagnosed and treated in childhood, a subset of patients remains undiagnosed until adulthood, when clinical presentation may include heart failure, pulmonary hypertension, or endarteritis [2]. Transcatheter closure using devices such as the Amplatzer™ Duct Occluder has become the standard of care for anatomically suitable PDAs, offering a less invasive alternative to surgery with excellent long-term outcomes [3].

Traditionally, aortography has been used as the primary imaging modality for procedural planning and device sizing during transcatheter PDA closure. However, aortographic measurement has limitations, particularly in adult patients, in whom the high flow velocity and variable ductal morphology may hinder accurate assessment of the minimal ductal diameter and length [4]. Furthermore, the oval or irregular shape of the PDA often seen in adults is not well characterized in two-dimensional angiographic views, potentially leading to suboptimal device selection, increased procedure time, or device replacement [5].

Advances in cardiovascular computed tomography (CT), including the development of high-resolution and photon-counting CT scanners, have enabled more precise, three-dimensional assessment of PDA morphology [5]. CT provides detailed cross-sectional imaging, facilitating more accurate measurement of the minimal and maximal diameters, length, and orientation of the ductus.

In this study, we aimed to investigate whether preprocedural CT improves the accuracy of device size selection compared to aortography alone in adult patients undergoing transcatheter PDA closure using the Amplatzer™ Duct Occluder.

## Methods

### Study population

**Fig. 1:**
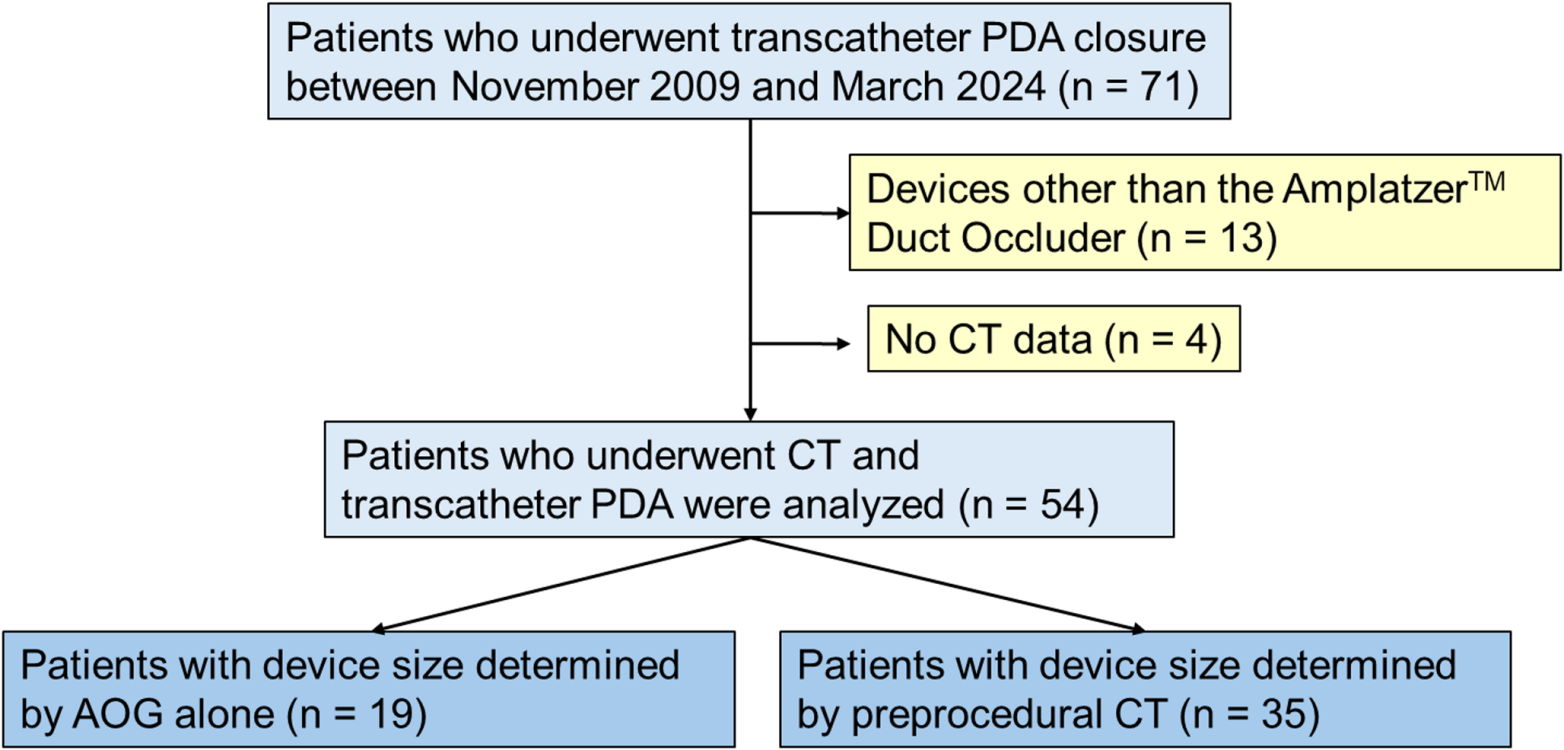
Flow diagram of patient selection. Of 71 adult patients who underwent transcatheter patent ductus arteriosus (PDA) closure, 54 who received preprocedural CT and were treated with the Amplatzer™ Duct Occluder were included. Device size was determined by intra-procedural aortography in 19 patients and by preprocedural CT in 35 patients. PDA, patent ductus arteriosus; CT, computed tomography; AOG, aortography

This retrospective, single-center study included consecutive adult patients who underwent transcatheter patent ductus arteriosus (PDA) closure at Okayama University Hospital between November 2005 and March 2024 (*n* = 71) (Fig. 1). Patients who were treated with devices other than the Amplatzer™ Duct Occluder (St. Jude Medical, St. Paul, Minnesota, USA) (*n* = 13) and those who did not undergo preprocedural CT (*n* = 4) were excluded. Ultimately, 54 patients who underwent both CT imaging and transcatheter PDA closure were included in the analysis. Although CT imaging was performed in all cases prior to the procedure, until 2010, the device size was determined intraoperatively based on aortography. Since then, however, device sizing has been determined based on preprocedural CT measurements of the PDA. Of the included patients, 19 patients were treated based on aortography alone, and 35 were treated based on preprocedural CT findings. This study was approved by the Ethics Committee of the Graduate School of Medicine, Dentistry, and Pharmaceutical Sciences at Okayama University (Okayama, Japan). The requirement for written informed consent was waived due to the retrospective nature of the study.

### Outcome measures

The primary procedural outcomes included the rate of device size exchange and procedure duration, which were compared between the aortography-alone group and the preprocedural CT group.

### Aortography

**Fig. 2:**
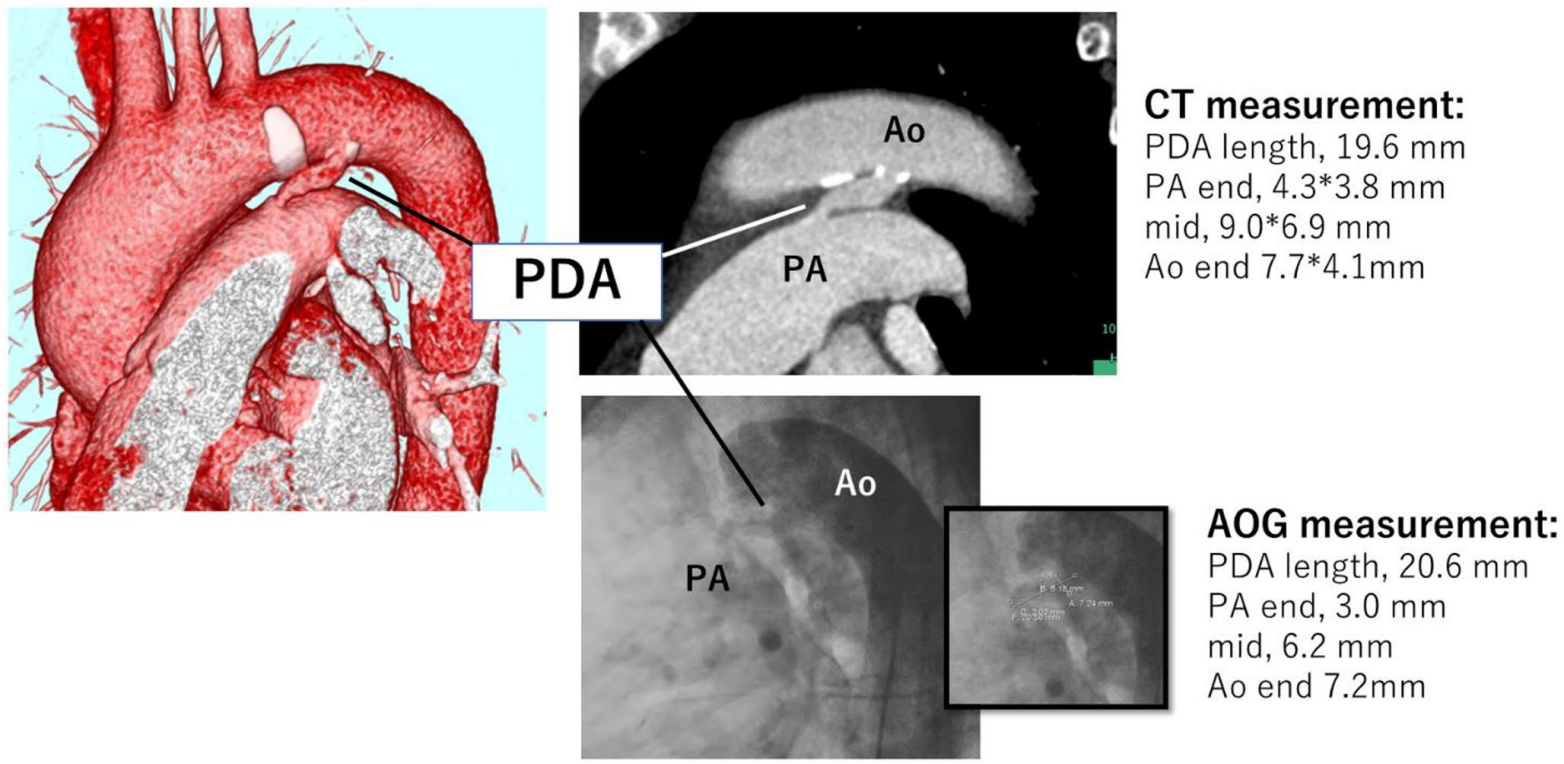
Representative case of PDA assessment using CT and aortography. Preoperative cardiac CT and intraoperative aortography revealed a stretched Krichenko type E PDA. The pulmonary artery end of the PDA, which is the most critical measurement for device selection, was measured larger on CT than on aortography. CT also revealed calcification on the aortic side of the PDA, suggesting the need for caution to avoid vascular injury during device deployment. Left: A three-dimensional volume-rendered CT image showing the PDA connecting the descending aorta and the pulmonary artery. Top right: CT image analysis showed the anatomical course and diameter of the pulmonary artery end (4.3 × 3.8 mm). Bottom right: Aortographic image showing measurements of the PDA at multiple levels: the aortic end (7.2 mm), the pulmonary artery end (3.0 mm), and the middle Sect. (6.2 mm), as well as the length (20.6 mm). PDA, patent ductus arteriosus. AOG, aortography. Ao, Aorta. PA, pulmonary artery

Aortographic images were acquired using a standard technique with a left anterior oblique angle of 90°, an injection rate of 15 mL/s, and a total of 30 mL contrast medium (Fig. 2). Based on the image, the length and the diameters at three locations, the pulmonary artery end, the aortic end, and the mid-section, were measured.

### CT image acquisition

**Fig. 3:**
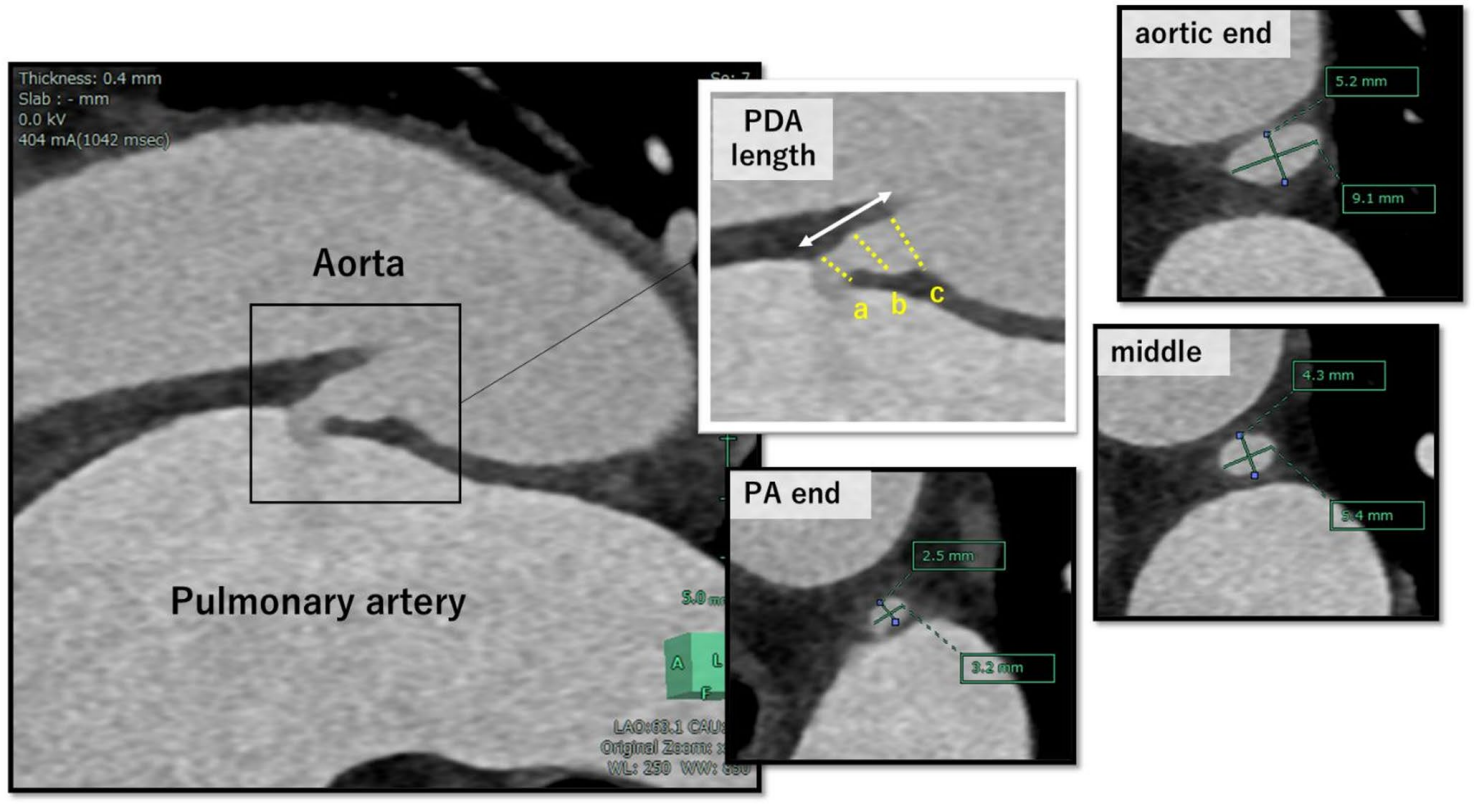
Representative CT images for PDA measurement. This figure illustrates the measurement of a PDA using three-dimensional CT analysis. In this representative case, the PDA length was 7.2 mm. The diameters at each point were as follows: 3.2 × 2.5 mm at the pulmonary artery end, 5.4 × 4.3 mm at the mid-section, and 9.1 × 5.2 mm at the aortic end. Additionally, CT evaluation revealed no significant coronary artery stenosis or anatomical issues with the access vessels relevant to transcatheter PDA closure

All CT scans were performed using either a 128-slice CT scanner (SOMATOM Definition Flash; Siemens Healthineers, Germany) or a dual-source photon-counting CT scanner (NAEOTOM Alpha; Siemens Healthineers, Germany). Scans were conducted using a standard coronary CT protocol. Double-oblique images, perpendicular to the PDA, were reconstructed using a dedicated workstation (SYNAPSE VINCENT; Fujifilm Medical, Tokyo, Japan). In the CT-based treatment, device size based on CT was determined preprocedurally by two cardiologists. To assess the PDA, its length and the diameters at three locations, the pulmonary artery end, the aortic end, and the mid-section, were measured. For diameter measurements, an oblique multiplanar reconstruction image was created along the long axis of the PDA. From this, a cross-sectional image perpendicular to the PDA’s central axis was obtained, and both the long and short diameters were measured at each of the three locations. (Figures 2 and 3).

### Actual procedure

Of all patients included in this study, 19 were treated based on aortography alone, and 35 were treated based on preprocedural CT findings. In the aortography-guided group, device size was determined by consensus between two cardiologists using intra-procedural aortographic images. Calibration of aortographic measurements was performed using the known outer diameter of a 4-Fr or 5-Fr pigtail catheter placed within the field of view, which served as an internal reference. In the CT-guided group, device size was determined by consensus between two cardiologists using preprocedural CT measurements prior to the catheter procedure. In both groups, device size was determined according to the manufacturer’s Instructions for Use. If the initially selected device was found to be undersized during implantation, it was replaced with a larger one.

### Simulation study

A simulation study was conducted using the angiographic and CT data of all patients. In this simulation, predicted device sizes were determined independently based on each imaging modality. The accuracy of these predictions was then evaluated by comparing them with the actual implanted device size, which was used as the reference standard. Device size simulations were performed by two experienced implanters (T.M. and T.A.), who were blinded to the actual implanted device size.

### Statistical analysis

Continuous variables are presented as mean ± standard deviation or median [interquartile rage] and were compared using the paired Student’s *t*-test or the Mann–Whitney *U* test, as appropriate. Categorical variables were compared using the chi-square test or Fisher’s exact test. Intra- and interobserver agreements for the measurements were assessed using Bland–Altman analysis and intraclass correlation coefficients (ICCs). A two-sided *p*-value < 0.05 was considered statistically significant. All statistical analyses were performed using SPSS version 29.0 (IBM Corp., Armonk, NY, USA).

## Results

### Actual procedure

Table 1 presents a comparison of patient characteristics between the aortography-guidance group and the preprocedural CT-guidance group. Among the 54 patients included, the mean age was 56 ± 19 years, and 84% were female. The PDA morphology was classified as Krichenko type A in 33 patients (61%), type C in 9 (17%), and type E in 12 (22%). Aortographic guidance was used in 19 patients and preprocedural CT guidance in 35 patients. Body surface area was significantly greater in the CT group compared to the aortography group. The PDA size measured during the aortography-guided procedure was 3.61 ± 1.21 mm, while the size determined by preprocedural CT was significantly larger (4.25 ± 1.06 mm, *p* = 0.049). The implanted device sizes were also significantly larger in the CT group (*p* = 0.01).

**Table 1:** Comparison of patients’ baseline and procedural characteristics between the aortography group and the preprocedural CT group

	All	Aortography guidance group	Preprocedural CT guidance group	*P* value*
	(*n* = 54)	(*n* = 19)	(*n* = 35)	
Age, years	56 ± 19	60 ± 17	54 ± 20	0.293
Male sex	9 (17)	1 (5)	8 (23)	0.097
NYHA classification *≥* II	25 (47)	9 (47)	16 (47)	0.983
Body surface area, m^2^	1.51 ± 0.18	1.42 ± 0.14	1.56 ± 0.18	0.006
Qp/Qs	1.61 ± 0.46	1.67 ± 0.58	1.58 ± 0.39	0.591
*Radiation dose in CT*				
CTDI, mGy	72.8 [35.1, 101.0]	70.7 [56.6, 83.9]	72.5 [20.5, 119.3]	0.755
DLP, mGycm	1508.3 [691.7, 2111.6]	1759.0 [970.8, 2806.0]	1505.7 [410.5, 2049.6]	0.302
Radiation dose in PDA closure, mGy	369.7 [262.1, 569.5]	392.9 [261.2, 575.1]	323.3 [232.3, 610.8]	0.874
*Implanted device size*				0.010
−6/4 mm	3 (6)	3 (16)	0 (0)	
−8/6 mm	12 (22)	5 (26)	7 (20)	
−10/8 mm	22 (41)	10 (53)	12 (34)	
−12/10 mm	14 (26)	1 (5)	13 (37)	
−14/12 mm	3 (6)	0 (0)	3 (9)	
Device size exchange	3 (6)	3 (16)	0 (0)	0.016
Procedure success	54 (100)	19 (100)	35 (100)	1.000
Complications	1 (2)	1 (5)	0 (0)	0.315
Procedure time, min	63 ± 12	70 ± 14	60 ± 9	0.003

Data are presented as mean ± standard deviation, median [25th percentile, 75 percentile], or number (%), as appropriate. *Comparison between the aortography alone and preprocedural groups. NYHA, New York Heart Association; Qp/Qs, pulmonary to systemic blood flow ratio. CTDI, computed tomography dose index; DLP, Dose length product

During the procedure, three patients in the aortography-guidance group required occluder replacement, whereas none in the CT-guidance group did. In all three cases, the initially selected device was undersized, and the procedure was successfully completed by upsizing the occluder. Procedure time was significantly longer in the aortography-guidance group than in the CT-guidance group (70 ± 14 vs. 60 ± 9 min, *p* = 0.003). One patient in the aortography-guidance group experienced transient hemolysis after device implantation. No major complications such as death, device embolization, or aortic dissection were observed in either group.

All patients in this study underwent preprocedural CT, with a radiation dose of 72.82 [35.1, 101.0] mGy in computed tomography dose index. The radiation dose during the PDA closure procedure was 369.8 [263.9, 558.2] mGy. The radiation dose during the PDA closure procedure did not differ between the aortography-guidance group and the CT-guidance group (*p* = 0.874).

### Simulation study

We next conducted a simulation study. Bland–Altman analysis of inter- and intra-assessor variability for PDA size measurements on CT and aortography showed limits of agreement of 0.094 ± 1.598 mm and 0.026 ± 0.444 mm, respectively, with no significant inter- or intra-observer differences. The ICC of the inter- and intra-observer agreement was 0.875 and 0.980, respectively.

**Fig. 4:**
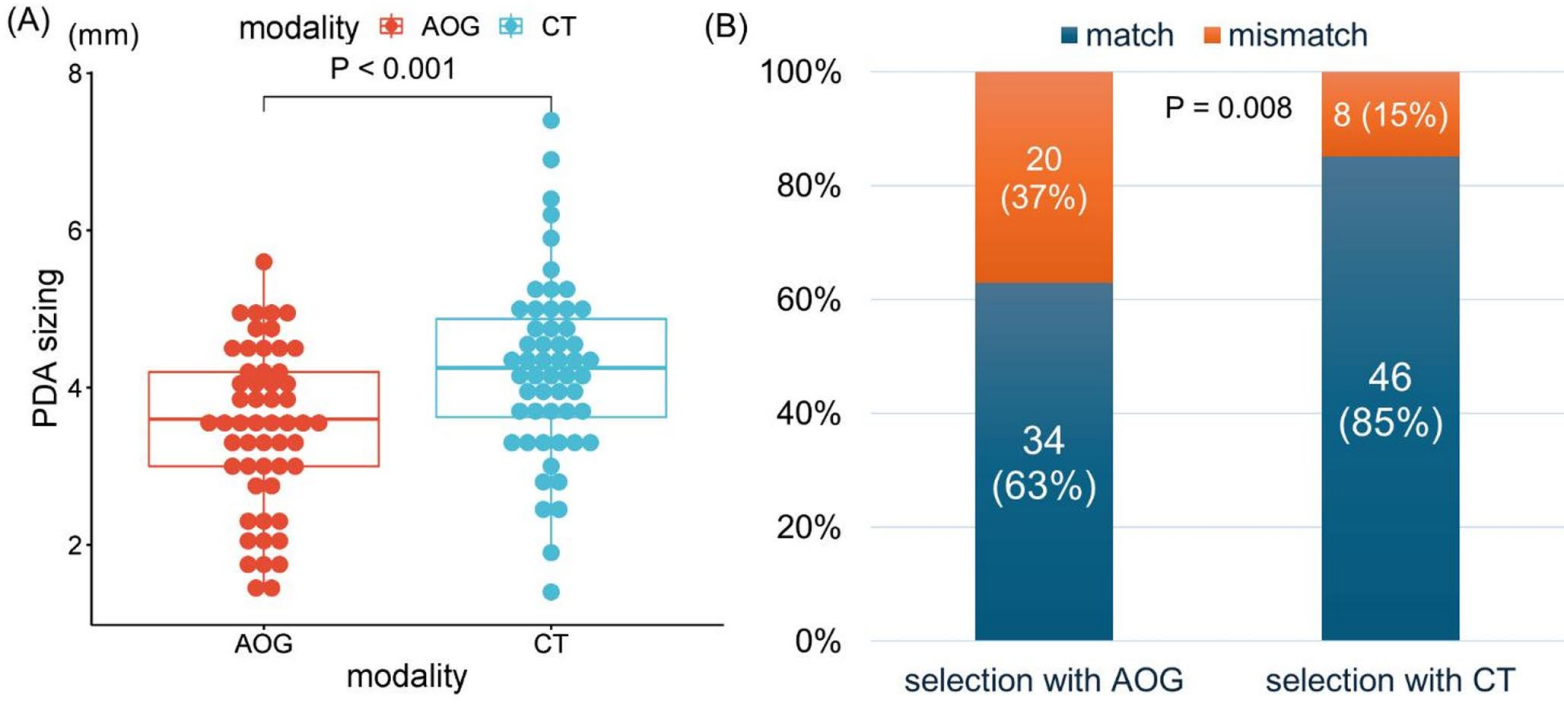
Results of the simulation study. (**A**) Comparison of PDA diameter at the pulmonary artery end measured by AOG vs. CT. CT measurements were systematically larger than AOG (3.48 ± 1.01 mm vs. 4.25 ± 1.15 mm, *p* < 0.001), with discrepancies ≥ 1 mm in 23 cases (43%). (**B**) Accuracy of occluder size prediction by modality: CT-based predictions matched the actually implanted device in 85% of cases vs. 63% with AOG (*p* = 0.008). Accuracy was defined as an exact match between the predicted occluder waist size and the device implanted; the implanted size served as the reference standard. AOG indicates aortography; CT, computed tomography.

PDA size at the pulmonary artery end was significantly underestimated on aortography compared with CT (3.48 ± 1.01 mm vs. 4.25 ± 1.15 mm, *p* < 0.001), with discrepancies ≥ 1 mm observed in 23 cases (43%) (Fig. 4A). The concordance rate between the imaging-based estimated device size and the actually implanted device was higher with CT (85%) than with aortography (63%) (*p* = 0.008) (Fig. 4B), indicating superior size prediction accuracy by CT.

## Discussion

In this retrospective single-center study, we demonstrated that preprocedural CT imaging was significantly more accurate than aortography in predicting the appropriate occluder device size in adults undergoing transcatheter PDA closure. Our findings revealed that CT-based planning was associated with a higher rate of correct device selection, reduced need for intra-procedural device replacement, and shorter procedure duration.

The superior performance of CT in device size prediction likely reflects its three-dimensional imaging capabilities, which allow precise visualization of the ductal structure perpendicular to the duct axis. Our data showed that PDA diameter at the pulmonary artery end was frequently underestimated by aortography, with more than 40% of cases showing a size discrepancy ≥ 1 mm when compared to CT measurements. Such underestimation can lead to the selection of undersized occluders, potentially resulting in procedural failure or the need for device exchange. Matsubara et al. further emphasized that CT-derived 3D modeling allowed accurate preselection of occluder size in all adult PDA patients in their simulation series, while angiography-based sizing required intra-procedural correction in half of the cases [5].

Our results are consistent with prior imaging studies demonstrating the advantages of CT for preprocedural planning in adult structural heart disease. Morgan-Hughes et al. initially highlighted the detailed morphologic assessment possible with ECG-gated MDCT in adult PDAs, emphasizing its potential in informing device strategy [4]. Moreover, Khajali et al. and Tanasan et al. each reported successful use of CT-based sizing in adult PDA closures, with high accuracy and reduced complications [6]. These findings support the integration of CT into clinical workflows for transcatheter PDA closure in adults, particularly when complex anatomy or diagnostic uncertainty is present.

From a practical standpoint, the additional cost of a preprocedural CT scan needs to be weighed against the potential savings it provides. In our study, device exchange was required in 16% of patients in the aortography group, whereas no exchanges occurred in the CT group. This corresponds to roughly six to seven patients needing CT to prevent one device exchange. Considering that each Amplatzer™ device represents a substantial expense and that a wasted device cannot be reused, the avoidance of even a single exchange may offset the cost of several CT examinations. Moreover, procedures guided by CT were, on average, 10 min shorter. Shorter procedures reduce catheter laboratory time, anesthesia exposure, and staff workload, which all translate into additional cost savings that go beyond the price of the device itself. Even if CT is not strictly cost-saving in every case, its ability to prevent unnecessary device use, reduce procedure duration, and provide additional information on ductal morphology and calcification supports its value as a cost-effective strategy, especially in older patients or those with complex anatomy.

In adult congenital heart disease, CT’s high spatial resolution and three-dimensional imaging capabilities enable comprehensive evaluation of morphologic complexity and calcification—features often underappreciated by angiography alone. For example, Saleh et al. describe how CT reveals complex postoperative anatomy and complications in patients with adult congenital heart disease [7]. Siripornpitak et al. further point out that CT can clearly delineate vascular stents and **c**alcified homografts, which are otherwise poorly visualized on MRI, thus improving procedural planning and safety profile [8]. Even in common anomalies such as PDA, Goitein et al. have shown that MDCT with multiplanar reformations can accurately depict calcification within the ductus, aiding in device selection and deployment strategy [9].

### Limitations

Several limitations of this study should be acknowledged. First, the study was retrospective and conducted at a single center with a relatively modest sample size, which may limit generalizability. Second, the study population was not randomized, and the choice of imaging modality used for planning was at the discretion of the treating physician, introducing potential selection bias. Third, although device selection was performed by experienced operators blinded to actual device size in the simulation component, interobserver variability may still have influenced the measurements. Lastly, long-term clinical outcomes were not assessed in this study.

## Conclusion

In adult patients undergoing transcatheter PDA closure, preprocedural CT imaging was associated with improved accuracy in occluder device size selection and reduced procedure time compared with conventional aortography. These findings suggest that CT may be a useful tool for procedural planning, potentially enhancing efficiency and safety. Further prospective studies are warranted to define its role in routine clinical practice.

## Data Availability

No datasets were generated or analysed during the current study.

## Abbreviations

PDA: Patent ductus arteriosus
CT: Computed tomography
